# Administration of Nebulized Ketamine for Managing Acute Pain in the Emergency Department: A Case Series

**DOI:** 10.5811/cpcem.2019.10.44582

**Published:** 2020-01-02

**Authors:** Jefferson Drapkin, Aidin Masoudi, Mahlaqa Butt, Rukhsana Hossain, Antonios Likourezos, Sergey Motov

**Affiliations:** Maimonides Medical Center, Department of Emergency Medicine, Brooklyn, New York

## Abstract

Ketamine administration in sub-dissociative doses in the emergency department (ED) results in effective pain relief in patients with acute traumatic and non-traumatic pain, chronic pain, and opioid-tolerant pain. This case series describes five adult ED patients who received nebulized ketamine for predominantly acute traumatic pain. Three patients received nebulized ketamine at 1.5 milligrams per kilogram (mg/kg) dose, one patient at 0.75 mg/kg, and one patient at 1 mg/kg. All five patients experienced a decrease in pain from the baseline up to 120 minutes. The inhalation route of ketamine delivery via breath-actuated nebulizer may have utility for managing pain in the ED.

## INTRODUCTION

Ketamine is a non-competitive N-methyl-D-aspartate/glutamate receptor complex antagonist that decreases pain by diminishing central sensitization, hyperalgesia, and “wind-up” phenomenon at the level of the spinal cord (dorsal ganglion) and central nervous system.^1^ Ketamine administration in sub-dissociative (SDK) dose (0.1–0.3 milligrams per kilogram (mg/kg)) in the emergency department (ED) results in effective pain relief in patients with acute traumatic and non-traumatic pain, chronic non-cancer and cancer pain, and opioid-tolerant pain by virtue of providing anti-hyperalgesia, anti-allodynia, and anti-tolerance.^2^,^3^ Two commonly employed administration strategies of SDK administration in the ED include an intravenous (IV) route (push-dose, short infusion, or continuous infusion), and intranasal route.^4^,^5^ However, in situations when IV access is unobtainable and/or mucosal atomization device is not readily available, nebulized routes of analgesic administration can be used. The nebulization of analgesics in the ED provides rapid, effective, and titratable analgesic delivery. It also results in less painful methods of analgesic delivery, minimizes analgesic toxicity and side effects (for example-opioids), and improves overall management of a variety of painful conditions in the ED.^6^

Nebulized administration of ketamine has been studied in the areas of palliative care, therapy for asthma, and acute postoperative management of sore throat.^7^–^9^ To our knowledge, there is no literature regarding analgesic efficacy and safety of nebulized ketamine’s role in managing acute painful conditions in the ED. The following cases describe five patients presenting to the ED of a tertiary medical center between May–June 2019 with acute painful conditions and receiving nebulized ketamine at three different dosing regimens of 0.75 mg/kg, 1 mg/kg, and 1.5 mg/kg via breath-actuated nebulizer.

## CASE SERIES

We describe five patients, ages 30–54, who presented to the ED with acute painful conditions: four patients with traumatic musculoskeletal pain, and one with abdominal pain (Table 1).

### Case #1

A 44-year-old man without prior medical history presented to the ED with one-day history of traumatic injury to his right wrist and hand with severe 8/10 pain and moderate swelling along the dorsal aspect of the right hand and wrist. Radiographs were negative for acute fracture or dislocation. The patient received only two doses of nebulized ketamine at 0.75 mg/kg with change in pain score from eight at the baseline to one at 120 minutes (min) (Table 2). The patient was discharged with a final diagnosis of wrist strain.

### Case #2

A 43-year-old woman without prior medical history presented to the ED with a chief complaint of 8/10 severe bilateral knee pain over the prior six months with pain radiating to both of her calf muscles and associated with stiffness. On physical examination the patient had moderate right patellar tenderness, no appreciable joint effusion, and no evidence of infectious/inflammatory processes. She received a single dose of nebulized ketamine at 1 mg/kg with a change in pain score from eight at baseline to zero at 120 min (Table 2). The patient was discharged with a final diagnosis of traumatic knee pain.

### Case #3

A 30-year-old man without prior medical history presented to the ED with severe, 8/10 pain to his left hand after slamming it with a car door. On examination, the patient was noted to have swelling and tenderness to palpation at the dorsal aspect of the left hand/metacarpal region with decreased range of motion at the second through fifth metacarpal joints and intact neurovascular status. He received nebulized ketamine at 1.5 mg/kg with change in pain score from eight at the baseline to two at 120 min (Table 2). The patient was discharged with a final diagnosis of hand contusion.

**CPC-EM Capsule**

What do we already know about this clinical entity?

Ketamine is used in the emergency department (ED) via intravenous, subcutaneous, and intranasal routes for managing acute painful conditions.

What makes this presentation of disease reportable?

This is the first description of the use of ketamine analgesia via breath-actuated nebulizer for adult ED patients presenting with acute painful conditions.

What is the major learning point?

Nebulized ketamine at doses of 0.75 milligrams per kilogram (mg/kg), 1 mg/kg, and 1.5 mg/kg alleviates acute pain in adult ED patients.

How might this improve emergency medicine practice?

Ketamine administration via breath-actuated nebulizer may serve as an additional non-invasive route for delivering analgesia in the ED.

### Case #4

A 54-year-old man without prior medical history presented to the ED with severe, 9/10 acute lower back pain after a fall at home that resulted in the patient landing on his back and subsequently sliding down 10 steps. On examination, he had prominent paraspinal tenderness at the lumbar region without swelling, ecchymosis, or laceration. He received nebulized ketamine at 1.5 mg/kg with change in pain score from nine at the baseline to zero at 120 min (Table 2). The patient was discharged with a final diagnosis of traumatic back pain.

### Case #5

A 38-year-old woman without prior medical history presented to the ED with chief complaint of severe 9/10 crampy abdominal pain and nausea and single episode of loose stool. On examination, the patient had prominent epigastric and periumbilical tenderness without rebound and guarding. She received IV famotidine and ketorolac without appreciable pain relief and subsequently received two doses of nebulized ketamine at 1.5 mg/kg with change in pain score from nine at the baseline to zero at 120 min (Table 2). The patient was discharged with a final diagnosis of viral gastroenteritis.

Three patients were male and two were female. None of the patients had absolute contraindications to ketamine such as allergy to ketamine, pregnancy, or history of schizophrenia. We used a standard verbal numeric pain-rating scale to evaluate patients’ pain scores at the baseline and at 15, 30, 60, 90, and 120 min.^10^ We used the Side-effects Rating Scale of Dissociative Anesthetics to describe side effects related to ketamine administration from baseline to 120 min.^11^

Three patients received nebulized ketamine via breath-actuated nebulizer at 1.5 mg/kg dose, one patient at 0.75 mg/kg, and one patient at 1 mg/kg. Two patients requested and received a second dose of nebulized ketamine. The dosages of nebulized ketamine (ordered and received) as well as baseline vital signs are presented in Tables 1 and 2. All five patients experienced a decrease in pain from baseline to 15 min, 30 min, 60 min, 90 min, and 120 min post medication administration (Table 2).

One patient experienced dizziness of modest intensity that was self-limited after 60 min and felt fatigued at 90 min. Another patient experienced a mood change, self-described as feeling “relaxed and happy,” at 15 min (Table 3). No patient experienced elevated heart rate and/or blood pressure during the observational period.

## DISCUSSION

In situations when intravenous access and/or mucosal atomization devices are not readily available, inhalation (nebulization) route might be considered for provision of timely and effective analgesia in the ED. However, safety and efficacy of ketamine in a nebulized form as an analgesic in the ED setting has yet to be shown.

Nebulized administration of ketamine has been studied in the areas of palliative care, asthma therapy, and acute postoperative management of sore throat.^7^–^9^,^12^,^13^ Five randomized trials have compared nebulized ketamine either in a fixed-dosing regimen (50 mg) or weight-based dosing (0.5 mg/kg, 1mg/kg, or 1.5 mg/kg) to placebo in reducing post-intubational sore throat. The average decrease of postoperative throat pain decreased by 44–50% without any major side effects.^7^–^9^, ^12^,^13^ Similarly, ketamine inhalation in healthy volunteers was easily tolerated and was not associated with oropharyngeal irritation, hypersalivation, stridor, laryngospasm, cough, dry mouth, hoarseness, dyspnea, tachypnea, aspiration, cardiac dysrhythmias, or desaturations.^14^,^15^

Pharmacokinetic properties of inhaled ketamine have not been studied broadly, but a single observational study evaluated pharmacokinetics of S-ketamine administered via nebulization to 12 healthy volunteers at doses of 0.35, 0.5, and 0.7mg/kg and inhalation duration of 20–40 min. The study demonstrated a time to maximum concentration of 15–22 min and a maximum concentration of 128ng/ml.^15^

In addition, based on the bioavailability of intranasal ketamine (25–50%) and oral ketamine (16–24%), the bioavailability to nebulized ketamine ranges between 20–40% of IV route.^16^,^17^ To date, this case series is the first to describe the use of nebulized ketamine via breath-actuated nebulizer at three different dosing regimens, with titration as needed for patients presenting to the ED with acute traumatic and non-traumatic painful conditions. The breath-actuated nebulizer (BAN) (AeroEclipse, Trudell Medical International, London, Ontario, Canada) provides smaller particles and greater dose delivery efficiency than continuous jet nebulizers. In addition, BAN possesses dual modes of action: 1) continuous aerosol generation; and 2) breath-actuated (in response to the patient’s inspiratory flow), ensuring that virtually no drug is lost to the environment.^18^,^19^ Furthermore, BAN has a potential to provide greater compliance and safer patient environment that may impact overall pain management, patients satisfaction, and length of stay in the ED.

All five patients had a decrease in pain from the baseline to 120 min with average change in pain score of 3.6 at 15 min, 4.6 at 30 min, 6.4 at 60 min, 7.6 at 90 min, and 7.8 at 120 min. Furthermore, only one patient experienced dizziness of modest intensity. While the descriptive nature of this report cannot be used to make any conclusion of safety and efficacy of nebulized ketamine in managing pain in the ED, the non-invasive route, titratability, and self-administration (by the patient) make this analgesic modality an attractive choice, especially when IV access is not readily available or unobtainable. There is a need for larger, dose-finding studies in a prospective, randomized fashion to fully evaluate the safety and efficacy of nebulized ketamine for analgesia in the ED.

## CONCLUSION

The inhalation route of ketamine delivery via breath-actuated nebulizer for managing pain in the ED may add an additional modality to the analgesic armamentarium of ED clinicians in providing rapid, effective, and non-invasive pain relief.

## Figures and Tables

**Table 1:** Characteristics of patients given nebulized ketamine.

Patient number	Age/sex	Chief complaint	Weight	Dosing mg/kg – actual dose^a^	Doses given	Total dose received^b^	Baseline vital signs			

							HR	BP	RR	SpO_2_
1	44M	Traumatic arm pain	70 kg	0.75 mg/kg – 55mg	2	55 mg	88	140/93	20	99
2	43F	Traumatic knee pain	67 kg	1.0 mg/kg – 65 mg	1	19.5 mg	78	112/58	16	100
3	30M	Traumatic arm pain	90 kg	1.5 mg/kg – 135 mg	1	108 mg	83	131/98	13	100
4	54M	Traumatic back pain	70 kg	1.5 mg/kg – 105 mg	1	105 mg	61	121/71	14	98
5	38F	Abdominal pain	65 kg	1.5 mg/kg – 100 mg	2	140 mg	72	116/72	15	97

a Actual dose: Indicates the per dose concentration the patient was meant to receive.

b Total dose received: Indicates the total amount a patient consumed after wastage.

*M*, male; *F*, female; *kg*, kilogram; *mg*, milligram; *HR*, heart rate; *BP*, blood pressure; *RR*, respiratory rate; *SpO_2_*, peripheral capillary oxygen saturation.

**Table 2:** Numerical pain scores.

Patient number	NRS pain over time					

	Baseline	15 min	30 min	60 min	90 min	120 min
1	8	6	5	5	1	1
2	8	5	3	2	0	0
3	8	5	4	2	2	2
4	9	6	4	0	0	0
5	9	2	2	1	1	0

*NRS*, numeric pain-rating scale; *min*, minutes.

**Table 3:** Severity of side effects using rating scale of dissociative anesthetics by patient and time.

Adverse effect	Time point	Level of severity			
		1-Weak	2-Modest	3-Bothersome	4-Very bothersome
Dizziness					
	15 min	--	Patient 2	--	--
	30 min	--	Patient 2	--	--
	60 min	--	Patient 2	--	--
	90 min	--	--	--	--
	120 min	--	--	--	--
Fatigue					
	15 min	--	--	--	--
	30 min	--	--		--
	60 min	--	--	--	--
	90 min	--	Patient 2	--	--
	120 min	--	--	--	--
Mood change					
	15 min	Patient 3	--	--	--
	30 min	--	--	--	--
	60 min	--	--	--	--
	90 min	--	--	--	--
	120 min	--	--	--	--

*min*, minutes.
